# Non-Destructive Characterization of Cultural Heritage Materials Using a Modified Acoustic Resonance Approach

**DOI:** 10.3390/ma19112291

**Published:** 2026-05-28

**Authors:** Filip Pantelić, Miloš Radomir Vasić, Marko Stojanović, Anja Terzić, Bojan Miljević, Snežana Vučetić

**Affiliations:** 1 Academy of Technical and Art Applied Studies, 24 Starine Novaka St., 11000 Belgrade, Serbia; filipp@viser.edu.rs; 2 Institute for Testing of Materials, Bulevar vojvode Mišica 43, 11000 Belgrade, Serbia; marko.stojanovic@institutims.rs (M.S.); anja.terzic@institutims.rs (A.T.); 3 University of Novi Sad, Faculty of Technology, Bulevar cara Lazara 1, 21000 Novi Sad, Serbia; miljevic@uns.ac.rs (B.M.); snezanap@uns.ac.rs (S.V.)

**Keywords:** non-destructive testing, stone, elements of earth architecture, mechanical strength, porosity, modulus of elasticity

## Abstract

This study explores non-destructive techniques for characterizing the mechanical properties of materials pertinent to cultural heritage, emphasizing the preservation of sample integrity. A modified acoustic resonance method (MARM), utilizing a two-microphone configuration, is introduced for the simultaneous, fully non-contact determination of dynamic elastic modulus and damping (loss factor). The method is validated through comparison with the impulse excitation technique (IET) and ultrasonic pulse velocity testing (UT). The approach is applied to two material categories exhibiting contrasting porosities: dense natural stone and highly porous unfired clay. Results demonstrate strong concordance among all methods for stone, confirming the reliability of non-destructive techniques for homogeneous materials. Conversely, unfired clay displays greater variability attributable to its heterogeneous and porous nature, alongside increased damping. This investigation reveals that conventional modulus–strength correlations are not directly applicable to unfired clay. To address this, a simplified strength estimation model incorporating the estimated elastic modulus and porosity is proposed. The model achieves improved alignment with experimental data and delineates applicability boundaries for porous materials. The presented framework facilitates consistent, non-destructive evaluation of mechanical properties, with notable implications for the assessment and preservation of cultural heritage materials.

## 1. Introduction

When performing material testing in architectural heritage structures or cultural heritage sites, one of the most important considerations is determining the best locations for material sampling. It is important to collect and conserve these samples in a way that minimizes the risk of damage to both the site and the materials involved [1,2,3]. In addition to conservation considerations, compliance with applicable legal and preservation laws is required [4,5]. Consequently, non-destructive testing procedures are strongly recommended to retain the integrity of the materials [6,7,8,9,10,11,12].

In terms of material characteristics, the elastic modulus is an important parameter in civil engineering [13,14]. It gives critical information on how construction materials behave under applied loads, allowing engineers to examine material behavior rapidly and accurately. Two principal types of elastic modulus, static and dynamic, offer distinct insights depending on the testing conditions and objectives. The static modulus is determined directly from stress–strain measurements, a method that is inherently destructive. Moreover, the static modulus and compressive strength exhibit a strong correlation and are integral to structural stability assessments. Standardized methodologies [15,16,17,18,19,20] are commonly used to determine the dynamic modulus. These techniques integrate elastic-wave velocity and density measurements and are non-destructive, because they use very modest strain amplitudes, ensuring the material remains within its elastic range during testing [21,22]. A systematic difference between dynamic and static elastic moduli was first identified in cement-based materials. This variation is due to discrepancies in measurement methodologies as well as the multi-phase nature of such materials. The strain rate applied during dynamic measurements is low, whereas it is higher during static testing, allowing for the detection of additional microcracking exclusively in static tests [23]. Lyndon et al. [24] and Popovics [25], in accordance with the British code of practice CP 110-1:1972 [26], primarily utilized equations to convert between static and dynamic elastic moduli. Furthermore, Eurocode 2 and ACI 318, widely adopted for concrete structural design in Europe and the United States, respectively, include equations and methodologies for predicting the compressive strength of concrete based on the elastic modulus [27,28]. Resonant frequency methods typically employ accelerometers as measuring sensors. However, their broader application is limited by factors such as susceptibility to environmental noise, signal distortion caused by sensor mass, and challenges related to sensor cost and attachment techniques to the sample [29,30,31]. Furthermore, the foundational measurement assumptions underlying ASTM E 1876-01 [15] and ASTM C215 [16] standards necessitate precise sample geometries, i.e., specifically prisms or cylinders, to minimize acoustic radiation and ensure accurate measurements. Additionally, the contact duration of the hammer impact and the sample’s length-to-diameter (L/D) ratio significantly influence test outcomes and, consequently, the estimation of concrete strength [32].

Cultural heritage samples frequently deviate from the idealized geometry prescribed by resonant frequency approaches. Their thickness and dimensions frequently result in resonances occurring at higher frequencies, often exceeding the critical frequency where acoustic radiation efficiency increases. This leads to insufficient minimization of acoustic radiation, potentially compromising measurement validity. In contrast, standard laboratory tests can be designed with optimized geometries that minimize acoustic losses and facilitate the separation of resonant frequencies, thereby enhancing measurement quality. However, for cultural heritage materials, testing must be done on samples in their original form, making such geometric optimization impossible [12,13,33]. To address this challenge, numerical simulations incorporating experimentally determined loss factors were developed using the COMSOL Multiphysics 4.3 software. These simulations model both internal losses (thermal and structural) within the sample and acoustic radiation losses, enabling an assessment of the reliability of signal time decay analysis for determining loss factors and defining the applicable frequency range for this approach [34,35].

The primary objective of this study was to analyze the time response of various vibration modes to determine properties (the loss factor) of heritage materials. This was achieved by tracking the decay of vibrations in the samples [36]. According to Piana et al. [37], energy loss in a material results from multiple loss mechanisms, including thermal losses, sound radiation into the surrounding air, and energy transfer to the supports on which the samples are mounted. The presented analysis focuses on inherent losses, which reflect energy dissipation mechanisms within the material itself and thus provide valuable information for nondestructive testing. Losses related to energy transfer to the supports predominate at low frequencies; however, above 45–50 Hz, the transmission of vibrational energy to the supports decreases significantly. Beyond the critical frequency, sound radiation into the surrounding air becomes prominent, coinciding with the acoustic wavelength in air becoming shorter than the elastic wave wavelength in the sample. Rindel [38] identified this frequency as the coincidence condition, where acoustic waves radiated into the air correspond to elastic waves propagating through the sample. The onset of acoustic radiation is therefore estimated by comparing characteristic structural dimensions, such as wavelengths and node spacing, with the corresponding acoustic wavelengths in air.

Accurate recording of all vibrational modes is essential to conduct the acoustic analysis. This requirement led to the study’s second objective, which was to propose a modified acoustic resonance method. This method combines impulse excitation with acoustic analysis of the sound response, where resonant frequencies are identified from the frequency spectrum of signals recorded by a microphone. The approach is grounded in the theoretical principles of the classical impulse excitation technique but enables fully non-contact measurement of the response without the need to attach sensors directly to the sample. As part of the methodology, a numerical model was employed to provide an initial assessment of oscillation behavior. A numerical model using COMSOL Multiphysics 4.3 was applied to assess oscillation behavior by analyzing the Very Near Field (VNF) [36], where sound pressure remains nearly constant close to the oscillation surface [39]. An eigenfrequency study estimated the natural resonance frequencies of samples. The recording method uses two microphones placed strategically to improve clarity, especially for torsional modes.

Recent research by Lara et al. [40] introduced a cost-effective, non-destructive method to estimate the static elastic modulus of early-age concrete using a smartphone microphone instead of accelerometers. This impact-resonance technique captures vibrations from hammer impacts on concrete cylinders, with a signal-processing algorithm extracting resonant frequencies from noisy audio. The microphone-based dynamic elastic modulus matched accelerometer accuracy, with less sensitivity to sensor coupling and easier on-site use. Khoso et al. [41] proposed a similar low-cost microphone method for concrete, identifying longitudinal frequency modes in fly ash concrete discs under various conditions. Frequency differences between microphone and accelerometer methods averaged less than 1%. By analyzing loss factors from reverberation time measurements, the elastic modulus can be expressed as complex quantities, broadening its physical interpretation. Following the formulation given in the study by O’Donnelli et al. [42], the real component corresponds to elastic energy storage, while the imaginary component accounts for dissipative losses. The elastic modulus, defined as the ratio of stress to strain, is purely real under quasi-static conditions where stress and strain remain in phase, indicating a fully elastic and lossless response. This study primarily addresses dynamic elastic moduli, where material damping introduces a phase lag between stress and strain.

Consequently, the third objective of the presented study was to predict the static elastic modulus by evaluating the complex modulus at a given frequency using measured damping. The first derivative of the fundamental dynamic modulus with respect to angular frequency was estimated based on simplified Kramers–Kronig relations [42,43]. After determining the static modulus, material properties were calculated and compared with values from the Lyndon and Popovics equations [24,25]. Compressive strength was also estimated and validated against destructive tests. Pabst et al. [44] review the relationship between elastic modulus and porosity in porous ceramics, noting conflicts between conventional models (Spriggs, Ishai–Cohen) and Hashin–Shtrikman bounds. The study revisits models like Coble–Kingery, Gibson–Ashby, and Hasselman, proposing a new formulation incorporating a critical porosity threshold linked to pore network percolation. This threshold marks a loss of pore connectivity, significantly affecting elastic behavior. Additionally, ceramics with similar pore structures follow a consistent “master curve” regardless of composition, highlighting the dominant role of pore morphology and connectivity. The current work builds on the empirical connections between Young’s modulus, shear modulus, and porosity revealed in research [44]. Given the large range of absolute values for Young’s and shear moduli in practically pore-free materials, the research focuses on their ratio. The porosity of each sample was estimated using a nonlinear fitting approach with a correlation constant ratio of 0.5 and experimentally determined values.

This study investigates two categories of materials with distinctly different porosities: a low-porosity natural stone and highly porous elements of earthen architecture, specifically manually prepared unfired clay bricks. The selection of stone and earthen (clay-based) materials was guided by their historical and cultural significance within both regional and global architectural heritage. Stone has been extensively utilized in sacral and monumental architecture, particularly in medieval Serbian monasteries, which are inscribed on the UNESCO World Heritage List. These structures predominantly employ low-porosity, mechanically robust stone materials. In contrast, earthen materials have a long history in vernacular architecture, particularly in the Vojvodina region, where construction techniques using locally available soils, such as adobe and rammed earth, have been frequently used. These materials are intrinsically porous and heterogeneous, which reflects both their composition and manual processing techniques. By focusing on these two material classes, the study captures the contrast between low-porosity, high-strength stone and highly porous, manually shaped earthen materials, allowing for a thorough investigation of the relationship between porosity and key physical-mechanical properties using non-destructive methods in the context of cultural heritage materials.

This research develops and validates a non-destructive acoustic framework for the reliable characterization of porous materials, facilitating its future application in the cultural heritage field without causing damage. The novelty of this study lies in the application of a two-microphone acoustic method for the simultaneous determination of dynamic elastic modulus and damping in solid materials, including its extension to unfired clay. This method allows for direct measurement of the loss factor within a single experimental setup. Furthermore, the study elucidates the role of damping in interpreting acoustic measurements and demonstrates that conventional modulus–strength conversion relationships are not applicable to unfired clay. To address these challenges, a damping-based interpretation criterion and a simplified compressive strength estimation approach based on predicted modulus and porosity are introduced. The applicability limits of the proposed methodology are also defined.

## 2. Materials and Methods

### 2.1. Materials Characterization

The stone material used in this study was classified as monzodiorite, an igneous plutonic rock with a modal composition of approximately 85% plagioclase. Plagioclase is the dominant feldspar, but it contains significant alkali feldspar (orthoclase). The major oxide composition was determined by X-ray fluorescence (XRF), showing 58.81% SiO_2_ and 16.95% Al_2_O_3_, with smaller amounts of Fe_2_O_3_ (6.61%), CaO (7.85%), MgO (2.83%), Na_2_O (4.58%), and K_2_O (4.70%). The loss on ignition (LOI) at 1000 °C was 0.67%. The clay used for the preparation of model samples is a clay mineral–rich material commonly used in the brick industry and in the production of porous building units. Its composition is dominated by quartz, muscovite, montmorillonite, illite, kaolinite, and hematite, which govern its pore structure and physical–mechanical behavior. The chemical composition of clay is as follows: SiO_2_ (57.74%), Al_2_O_3_ (28.67%), Fe_2_O_3_ (1.04%), CaO (0.71%), MgO (1.14%), Na_2_O (0.57%), and K_2_O (2.44%), and LOI at 1000 °C (7.69%).

Chemical analysis of raw materials was performed using energy-dispersive X-ray fluorescence (ED-XRF) on a Spectro Xepos system (Spectro Scientific Analytical Instruments, Chelmsford, MA, USA). The XRF apparatus is equipped with a 50 W, 60 V X-ray tube featuring a binary Co/Pd alloy thick target anode. The X-ray tube works in a combined polarized and direct excitation mode. Characteristic radiation emitted by the elements within the samples was detected using a silicon drift detector cooled by a Peltier system. Pulverized samples with a median particle size below 63 μm were utilized for the analyses. Prior to pulverization in a Herzog vibratory disc mill (HERZOG Maschinenfabrik, Osnabrück, Germany), raw materials were dried at 100 °C in a laboratory dryer. Loss on ignition (LoI) was determined at 1000 °C using a laboratory furnace.

Although standardized methods [45,46,47,48,49,50,51,52,53,54] were employed, the study focuses primarily on non-destructive techniques and their relation to key physico-mechanical properties. Therefore, detailed material characterization is not presented. Instead, emphasis is placed on selected properties, such as porosity, water absorption, and strength-related parameters, that are directly correlated with results obtained using MARM, IET, and UT. This approach enables the evaluation of structure-property relationships while preserving sample integrity, which is essential for cultural heritage materials.

The models of earthen architecture elements were prepared by manual shaping, mimicking traditional fabrication techniques of earthen architecture and promoting the development of a heterogeneous and relatively high-porosity structure (dimensions 40 × 40 × 160 mm). All tests on the earthen architecture elements were performed after a curing period of six months. Specifically, the samples were stored under laboratory conditions for six months following shaping and prior to testing, allowing for stabilization of their microstructure and physico-mechanical properties. Stone samples were cut in the laboratory from the supplied stone slabs to dimensions of 300 × 50 × 50 mm. The samples were then dried to constant mass.

### 2.2. Non-Destructive Testing Methods

The focus of this study was on non-destructive testing methods, while destructive tests were used only as reference measurements. The applied techniques include the Modified Acoustic Resonance Method (MARM), based on a two-microphone acoustic measurement setup. For validation purposes, the Impulse Excitation Technique (IET) and Ultrasonic Testing (UT) were also employed.

The modified acoustic resonance method (MARM) required that the sample be excited by a short mechanical impulse, with the acoustic response recorded by a microphone at a controlled distance. As part of the methodology, a numerical model was employed to provide an initial assessment of the oscillation behavior. Numerical modeling was performed using COMSOL Multiphysics version 4.3. The simulations were based on the Solid Mechanics module, and an eigenfrequency study was conducted to estimate the natural resonance frequencies of the tested samples preliminarily. The numerical model incorporated the precise geometric dimensions and densities of the samples, which were experimentally determined from mass and volume measurements.

Each sample was modeled as a homogeneous, linear elastic solid. Initial values of the elastic constants, namely Young’s modulus and Poisson’s ratio, were taken from literature for clay (*E* = 10 GPa and *ν* = 0.25) and stone (*E* = 60 GPa and *ν* = 0.23). The finite element method was used to solve the governing equations under free–free vibration conditions. Since the objective of the numerical analysis was limited to identifying resonance frequencies, an eigenfrequency study was deemed sufficient. The computational domain was discretized. A three-dimensional finite element mesh comprising 2348 and 5281 elements was used for the stone and clay samples, respectively. Physics-controlled meshing parameters were applied. Material damping and fluid–structure interactions were neglected, as their influence on the results was considered negligible within the scope of this study. Numerical simulations yielded the distribution of resonances, which guided the experimental measurements.

**Figure 1:**
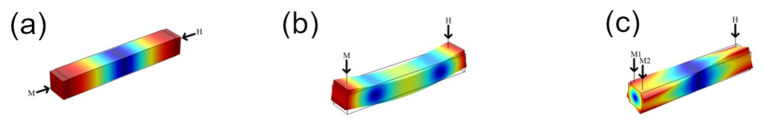
Microphone positions and numerically obtained resonance distribution: (**a**) NTI M 4260 measurement; (**b**) VFN—longitudinal resonance; (**c**) torsional mode.

Special consideration was given to the sample supports to minimize their impact on the resonant frequency measurements. Small rubber mounts, approximately 5 × 10 × 50 mm in size, were placed near the nodal lines corresponding to each oscillation mode. The experimental procedure continued with the excitation of the sample’s longitudinal oscillation mode. This was achieved by delivering an impulse impact along the longitudinal axis using a wooden hammer. An NTI M 4260 measurement microphone captured acoustic responses at approximately 2 mm from the free end of the sample, as shown in Figure 1a.

This methodology uses the characteristics of the Very Near Field (VNF) for assessment of vibrational properties [36]. The sound pressure amplitude attenuates minimally across small spatial distances near the oscillation surface [39]. As a result, slight differences in microphone placement distances (between 2 and 4 mm) have no significant effect on observed signal levels. The captured signals were examined using Fourier transformation to produce the frequency spectrum. A dominant longitudinal resonance peak, accompanied by several secondary resonance frequencies, was registered. Based on the numerical model, the longitudinal resonant frequency was identified in the measured spectrum. By using the identified longitudinal resonance frequency, together with the known sample length and material density, the longitudinal Young’s modulus was calculated as follows, where ρ, *L*, and *f* denote the density, sample length, and the frequency of the first longitudinal resonance, respectively. This experimentally derived modulus was subsequently incorporated into the COMSOL model, enabling refinement of the numerical simulation and enhancing its predictive accuracy for other resonant modes.

Following calibration of the model based on the longitudinal resonance data, the investigation proceeded to measure the flexural oscillation modes. The samples were excited by an impact delivered near the beam edge from above, while the microphone was placed on the opposite side at a similar distance in VNF (Figure 1b). The first flexural mode is dominant in the spectrum of the recorded signals. Although higher-order flexural modes were detected too, they were excluded from the scope of the present analysis.

The torsional mode was measured by positioning a microphone at the sample’s corner from the top, while excitation was induced by striking the diagonally opposite corner (Figure 1c). In a clay sample, the acoustic response associated with torsional modes is notably weaker than for other modes and is therefore difficult to identify in the measured spectra. Numerical simulations are helpful in this process; however, this type of excitation also induces flexural modes, which are typically stronger than the torsional mode and may obscure its identification. To validate the torsional mode identification, the sample was rotated 90° around its longitudinal axis, and the measurements were repeated. The torsional resonance frequency stayed constant, however the flexural resonance shifted when the width and thickness parameters of the sample changed.

**Figure 2:**
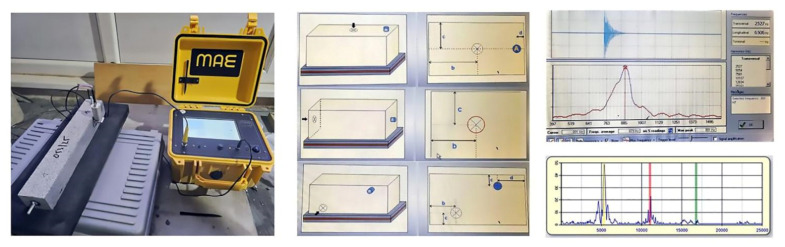
IET experimental setup and numerically obtained resonance distribution.

In addition to the modified acoustic resonance method, the standard impulse excitation technique with an accelerometer as a sensor (IET) and the ultrasonic wave (UT) method were also used. Measurements on stone and clay samples were conducted in accordance with ASTM C215. Mechanical excitation was achieved via a light hammer. The mechanically induced inputs generate a transient elastic response in the sample, which is subsequently detected by the highly accurate MAE C 311R device (manufactured by M.A.E. s.r.l., Isernia, Italy) which integrates a high-sensitivity piezoelectric acoustic sensor, an advanced signal conditioning unit, and a high-resolution digital data acquisition system. For each sample, five multiple measurements were systematically performed, and key waveform characteristics, including amplitude levels, signal duration, and temporal features, were detected. The experimental setup is illustrated in Figure 2.

**Figure 3:**
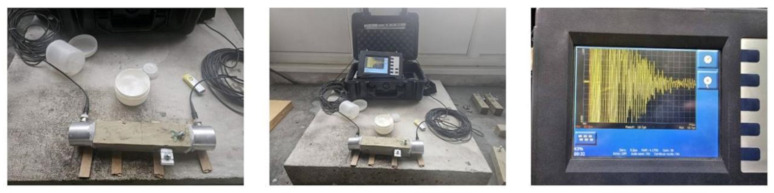
UT experimental setup.

The UT method measures the speed of longitudinal elastic waves through the sample. Using this wave speed, along with the material’s bulk density and an estimated or measured Poisson’s ratio, the dynamic modulus of elasticity was calculated. The testing was carried out in accordance with SRPS U.M1.042:1998. The image of the experimental setup is provided in Figure 3.

After the initial frequency analysis, a time response analysis was performed using the Schroeder curve method. Reverberation times were measured within frequency bands around the resonant frequencies and used to find loss factors for longitudinal, flexural, and torsional modes. This method is similar to that used in room acoustics, where T60 represents the time it takes for the sound energy to decrease by 60 decibels. In practice, owing to background noise and restricted stimulation strength, decay is frequently recorded over smaller ranges, such as 10, 20, or 30 dB, and then extrapolated to determine T60.

In this investigation, the impact hammer delivered the excitation, resulting in impulse responses of limited amplitude. As a result, the full 60 decibels of decay could not be measured. Instead, the T10 approach was utilized, which examines the time required for the energy to decay from −5 dB to −15 dB relative to the maximum. The reverberation time was computed using the Schroeder curve, which is formed by integrating the squared impulse response backwards, demonstrating how vibrational energy declines over time.

Since the experiments involved small samples, the reverberation times are much shorter than in the room acoustics. The energy decays quickly, resulting in steep Schroeder curves and small integral values. This makes the T10 interval a reliable way to estimate damping properties. Due to the very small sample dimensions and rapid signal decay, reliable estimation of a full 60 dB decay was not experimentally feasible; therefore, the T10 approach was adopted using the standard extrapolation procedure. The loss factor was calculated using Equation (1) [37]:

$$s\left(t\right)=\int_{t}^{\infty }h^{2}\left(\tau \right)d\tau ;\;$$

where h(t) denotes the measured impulse response;

(1) $$\eta =\frac{2.2}{fT_{10}}$$

To understand how this damping compares to the material’s intrinsic energy losses, a numerical analysis with COMSOL Multiphysics was applied. A harmonic frequency-domain analysis was used to model energy dissipation, with material damping provided by an isotropic loss factor of 0.04 for clay and 0.003 for stone, assuming linear viscoelastic behavior. To replicate acoustic radiation, the sample was connected to an air domain via a fluid–structure interaction interface. The acoustic field was modeled using the Pressure Acoustics module, and non-reflective boundary conditions precluded fake reflections.

The internal energy loss was calculated by integrating the volumetric loss density over the sample’s volume across the frequency range. The radiated acoustic power was found by integrating the acoustic energy flux over the air domain’s boundary surfaces. This flux, based on the product of acoustic pressure and particle velocity, allowed us to compare the internal dissipation with the energy lost through sound radiation.

Using the identified resonant frequencies, Young’s moduli associated with the longitudinal and flexural oscillation modes were computed, along with the shear modulus derived from the torsional mode. Poisson’s ratio was subsequently determined from these elastic parameters. The resulting elastic constants represent effective values influenced by the porosity of the unfired clay. Both Young’s modulus and shear modulus exhibited a decreasing trend with increasing porosity; therefore, the experimental data were fitted using an Archie-type power-law relation to provide a compact empirical description given by Pabast [44].

(2) $$E=E_{0}{\left(1-\phi \right)}^{n};G=G_{0}{\left(1-\phi \right)}^{k}$$

Here, *E*_0_ and *G*_0_ denote the Young’s modulus and shear modulus of an almost pore-free material, *ϕ* is the porosity, while *n* and *k* are fitting parameters describing the sensitivity of Young’s modulus and shear modulus to porosity, respectively. Literature reports a range of exponents for similar porous materials. In this context, *n* = 2 corresponds to the idealized Coble–Kingery relation and represents a lower bound. At the same time, an Archie-type fit with *n* = 2.61 reflects a typical value observed for ceramics with matrix-inclusion microstructures. In the present study, an intermediate value of *n* = 2.5 was adopted. For the shear modulus, a lower exponent, *k* = 2, was adopted, reflecting the generally weaker sensitivity of shear stiffness to porosity compared to Young’s modulus. Due to the pronounced variability of the absolute values of *E*_0_ and *G*_0_, the analysis was further based on the ratio of the two relations, *E/G*, as the ratio of *E*_0_/*G*_0_ seems to be more stable.

To improve stability, assuming and using experimentally measured values and a nonlinear fitting procedure enabled the estimation of porosity for each sample. The analysis based on the *E*/*G* ratio demonstrated greater robustness than approaches relying on individual elastic moduli.

Based on the loss factors obtained from the reverberation time analysis, the elastic moduli can be expressed as complex quantities, thereby extending their physical interpretation. In this framework, the elastic modulus is written as in [42], where E*(ω) (Equation (3)) is the complex dynamic modulus, *E*_d_(ω) is the measured dynamic modulus, *E*_l_(ω) is the loss modulus, and *η*(ω) is the loss factor.

(3) $$E^{*}\left(\omega \right)=E_{d}\left(\omega \right)+iE_{l}\left(\omega \right)=E_{d}\left(\omega \right)(1+i\eta \left(\omega \right))$$

The real part corresponds to the elastic energy storage, while the imaginary part represents dissipative losses. The elastic modulus is the ratio of stress to strain. When the modulus is purely real, stress and strain are in phase, indicating a fully elastic, lossless response. This assumption is valid only under quasi-static conditions. In the present study, the focus is on dynamic elastic moduli, in which material damping induces a phase lag between stress and strain. Although the imaginary part of the elastic modulus is small, it indicates the presence of energy dissipation mechanisms and results in a measurable phase difference. For the analyzed stone sample, damping is low, whereas for the dried clay sample, losses are more pronounced.

An additional insight into the relationship between the real and imaginary components of the elastic modulus can be obtained through the Kramers–Kronig relations, which link the frequency-dependent storage and loss components of a causal linear system [43]. For the investigated materials, the dynamic elastic modulus is expected to increase with frequency, which is consistent with general viscoelastic behavior.

In the current experiments, the elastic modulus (Equation (4)) is calculated at discrete resonance frequencies that correspond to separate vibration modes. A complicated elastic modulus can be calculated at a specific frequency using the measured damping. Ouis reported that, given simplified assumptions derived from the Kramers-Kronig relations, the first derivative of the real part of the dynamic modulus with respect to angular frequency can be determined locally [42].

(4) $$E_{l}\approx \frac{\pi }{2}\omega \frac{dE_{d}(\omega )}{d\omega }$$

This allows for a rough estimate of the static elastic modulus by extrapolating the frequency-dependent trend towards lower frequencies. Although such an extrapolation presupposes consistent functional behavior across the whole frequency range, which may not be precisely correct, it provides a good qualitative assessment. Compared to stone, where losses are minimal and frequency-dependent, dried clay has substantially larger damping. As a result, a more prominent fluctuation of the elastic modulus towards lower frequencies, and possibly toward the static regime, can be expected.

### 2.3. Destructive Testing Methods

The mechanical properties of earthen architecture elements (clay-based materials) and stone samples were evaluated using flexural and compressive strength. The hydraulic press with a capacity of 3000 kN (Matest, Treviolo, Italy) was employed. Mercury intrusion porosimetry was measured using a Micromeritics AutoPore IV 9500 Mercury Porosimeter (Micromeritics Instrument Corporation, Norcross, GA, USA). Due to the significant heterogeneity of the earth’s elements and a small sample size, measurements were taken in triplicate to assure reproducibility. In the case of stone samples, based on their homogeneity, these measurements were performed on two separate samples. As a result, three independent samples of both stone material and clay-based materials for earth architecture were examined using mercury intrusion porosimetry.

## 3. Results and Discussion

The initial section represents a comprehensive analysis of the results obtained from non-destructive testing methods and their application in accurately estimating the mechanical properties of the materials under investigation. The study focuses on evaluating key parameters such as the dynamic elastic modulus and damping characteristics (expressed as the loss factor), which are derived through advanced techniques including the Modified Acoustic Resonance Method (MARM), the Impulse Excitation Technique (IET), and Ultrasonic Testing (UT). Detailed examination of these parameters provides critical insights into the material behavior and performance without compromising the integrity of the samples.

### 3.1. Resonance Frequency

**Figure 4:**
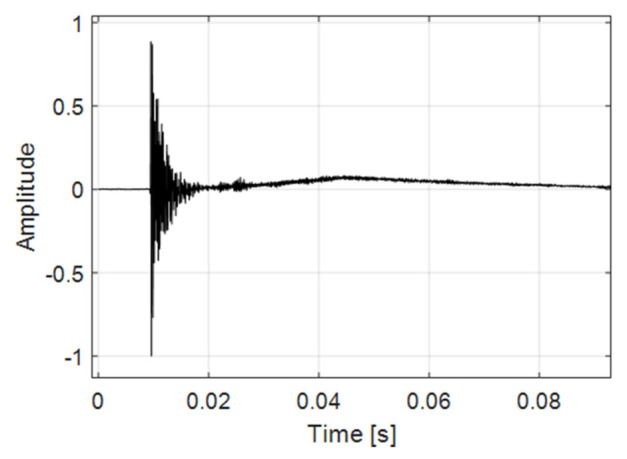
Representative impulse response in the time domain and the corresponding wave shape.

**Figure 5:**
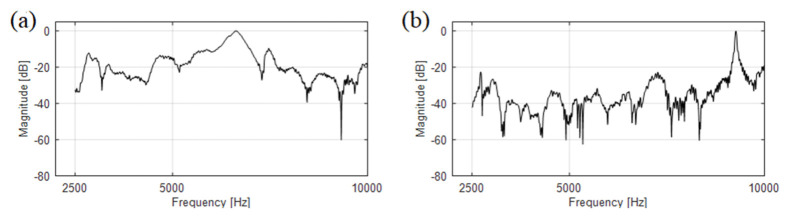
Spectra of impulse responses of samples excited in longitudinal direction: (**a**) clay; (**b**) stone.

Figure 4 depicts a representative impulse response captured with a measuring microphone. Figure 5 also shows the spectra of the impulse responses for dried clay and stone samples that were excited in the longitudinal direction. The Fourier transformation was used to assess the dynamic responses of the samples under investigation. The obtained spectra aided in the identification of resonance frequencies associated with longitudinal, flexural, and torsional vibration modes, which served as the foundation for further investigation.

Numerical models were developed for stone (*E* = 60.00 GPa, *ν* = 0.23) and dry clay (*E* = 10.00 GPa, *ν* = 0.25) based on the measured mass and dimensions of the samples. These properties of a material correspond to typical values reported in the literature and were employed in the initial iteration of the modeling process. Experimentally obtained resonance spectra were subsequently compared with resonance frequencies predicted numerically through finite element analysis. In the numerical simulations, the investigated materials were modeled as homogeneous and isotropic continua with effective elastic properties obtained experimentally. The influence of porosity was not modeled explicitly through discrete pores but was incorporated indirectly through the experimentally determined effective elastic and damping properties.

Table 1 presents a summary of the experimentally measured resonance frequencies alongside their numerical counterparts for the stone and clay samples, respectively. For both materials, the initial numerical iteration, based on assumed elastic parameters, exhibited noticeable discrepancies from the experimental values; however, it was essential to establish a predictive framework indicating the expected resonance regions. According to numerical simulations for both stone and clay, the longitudinal resonance frequency is anticipated to be significantly higher than the flexural and torsional resonances. In the spectra shown in Figure 5, the longitudinal resonance consistently displays the highest frequency in the recorded impulse response for longitudinal excitation. Notably, the dry clay sample exhibits higher damping, as evidenced by its less pronounced resonance peak compared to the stone sample.

Using the detected longitudinal frequencies, the modulus of elasticity was calculated and subsequently updated in the numerical model. This refinement led to a second numerical iteration that yielded resonance frequencies in close agreement with the experimental measurements, facilitating the identification of longitudinal, flexural, and torsional modes. A detailed analysis revealed the presence of closely spaced resonances within the flexural vibration range for the clay samples. The occurrence of two proximate flexural resonances is attributed to the comparable dimensions of sample width and thickness, resulting in flexural oscillations about two orthogonal axes with similar stiffness characteristics.

For the stone sample, realistic elastic parameters produced a flexural resonance at 2759 Hz, corresponding to flexural vibration about the sample’s minor axis. The numerical model also predicts a second flexural mode about the major axis at 2960 Hz. The identification of this mode is critical, as its higher frequency may otherwise be misinterpreted as a different vibration pattern. In particular, if this frequency falls within the torsional mode range, modal coupling may occur. Such coupling can alter the experimentally measured resonance frequencies and influence the observed damping. When two resonant modes become coupled, they effectively behave as a single system, exchanging vibrational energy and resulting in mixed damping characteristics.

The presence of two closely spaced flexural modes may lead to partial modal overlap and mixed damping characteristics. However, the resonance peaks remained sufficiently separated to enable independent peak identification using narrow-band spectral analysis around each resonance frequency. Although the modal coupling effect was not quantified separately, its influence on the measured loss factor of the stone samples is considered limited, particularly in comparison with the significantly higher damping observed for unfired clay.

**Figure 6:**
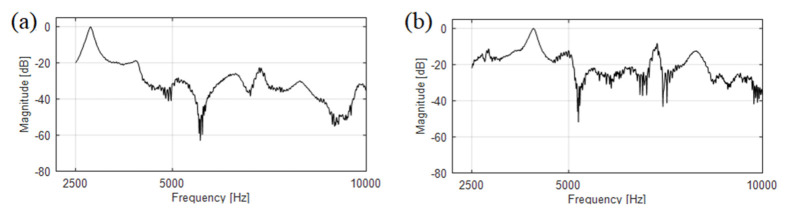
Comparison of torsional-mode spectra obtained using: (**a**) single and (**b**) dual microphone recording configurations.

Flexural modes are typically straightforward to identify. In Figure 6a, spectra are presented for the impulse response of a sample excited at its corner to induce torsional oscillation. Even under these conditions, the flexural mode is also excited and predominates in the spectra. The implementation of a dual-microphone recording configuration enabled clearer identification of torsional resonances in clay samples (Figure 6b), where the acoustic response of torsional modes is inherently weak. This method enhanced the separation of overlapping resonances and facilitated reliable identification of torsional modes, which could be advantageous when employing automatic mode detection algorithms in signal processing.

Torsional and flexural oscillations are governed by distinct deformation mechanisms and may therefore exhibit different damping constants. These differences are particularly significant as they provide insights into internal material characteristics such as microstructure and porosity. Density and geometric characteristics of tested samples are provided in Table 2.

Table 3 presents the average resonant frequencies of the model samples, measured using both the modified acoustic resonance method (MARM) and the impulse excitation technique (IET). The results are very consistent across all three vibration modes: longitudinal, flexural, and torsional. For the stone, the longitudinal frequency measured with MARM (9225 Hz) is slightly lower than that with IET (9574 Hz), and the flexural and torsional frequencies are very close. A similar pattern is observed with the models of earth elements, indicating that MARM yields results comparable to those of the traditional IET method.

### 3.2. Damping and Dynamic Elastic Parameters

**Figure 7:**
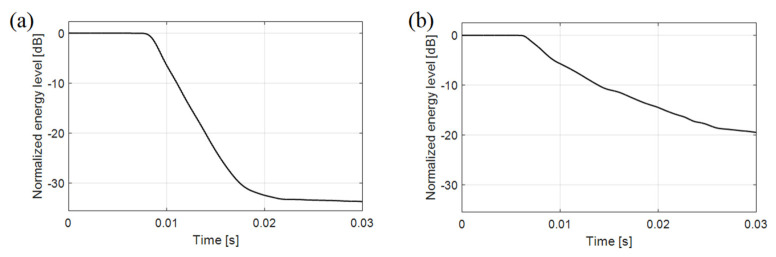
Schroeder curve obtained from the impulse response of sample oscillating in the torsional mode: (**a**) clay and (**b**) stone.

Reverberation times obtained from Schroeder curve analysis (as seein in Figure 7) were utilized to calculate damping factors for longitudinal, flexural, and torsional vibration modes.

The loss factor was calculated using Equation (1) [37]. The loss factor was determined for the investigated samples as well as the longitudinal, torsional, and flexural modes of oscillation, as shown in Table 4. The stone sample exhibited extremely low damping levels, with loss factors ranging from 0.0027 to 0.0063, depending on the vibration mode. In comparison, the model sample of earth architecture had substantially greater loss factors, typically about 0.04, indicating moderate internal friction.

In oscillating systems, the introduction of damping typically results in a reduction in natural frequencies. However, for the samples examined in this study, the damping-induced shifts in resonance frequencies were minimal and thus excluded from the subsequent calculations of elastic constants.

As previously noted, the measured loss factor does not exclusively reflect intrinsic material losses, as a portion may arise from acoustic radiation. Therefore, it is essential to assess the extent of this effect, since acoustic radiation can contribute to the measured damping and must be accounted for in both materials under investigation. The efficiency of radiation is influenced by the sample’s geometry, resonant frequencies, and the ratio between structural dimensions and acoustic wavelengths in both the material and the surrounding air. For the clay sample, flexural modes occur near 2.8 kHz. At this frequency, half the acoustic wavelength in air is approximately 6 cm, while the wavelength of flexural waves within the sample is about 9.5 cm. Notably, the distance between vibration nodes, which governs acoustic radiation, is roughly 17% less than half the wavelength in the sample, measuring approximately 7.9 cm [36].

Regarding torsional modes in both materials, acoustic radiation is limited due to mode shapes where adjacent segments oscillate in opposite phases and are situated in close proximity. For longitudinal oscillations, the smallest transverse faces of the sample serve as the radiating surfaces. The acoustic wavelengths in air for samples vibrating in the first longitudinal mode are approximately 3.7 cm for stone and 5.3 cm for clay, which is sufficient to facilitate radiation given the size of these surfaces [55]. Based on these considerations, it can be concluded that both flexural and longitudinal vibration modes occur within frequency ranges where acoustic radiation may take place. To quantify the contribution of this effect relative to intrinsic material losses, a numerical analysis was conducted using COMSOL Multiphysics.

**Figure 8:**
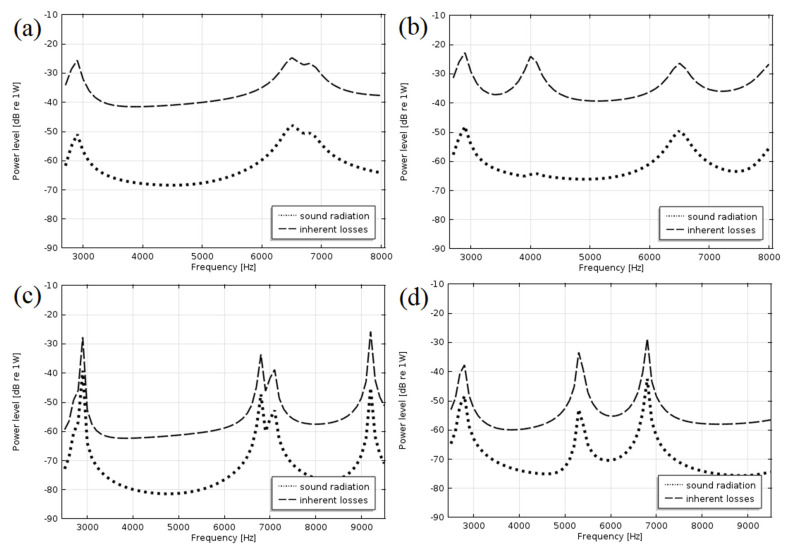
Clay: longitudinal (**a**); transversal/torsional (**b**). Stone; longitudinal (**c**); transversal/torsional (**d**).

Material damping was incorporated into the existing model via an isotropic structural loss factor, with values set at 0.04 for clay and 0.003 for stone. The analysis was performed in the frequency domain to capture response characteristics across the relevant frequency range. Results are given in Figure 8. The internal dissipated power was calculated by integrating the volumetric loss density over the entire sample throughout the analyzed frequency range. Concurrently, the radiated acoustic power was determined by integrating the acoustic energy flux over all six boundary surfaces of the surrounding air domain. This methodology allows a direct comparison between the internally dissipated energy and the energy lost through acoustic radiation. Additional processed experimental datasets and supplementary acoustic measurement data are provided in the Supplementary Materials.

**Figure 9:**
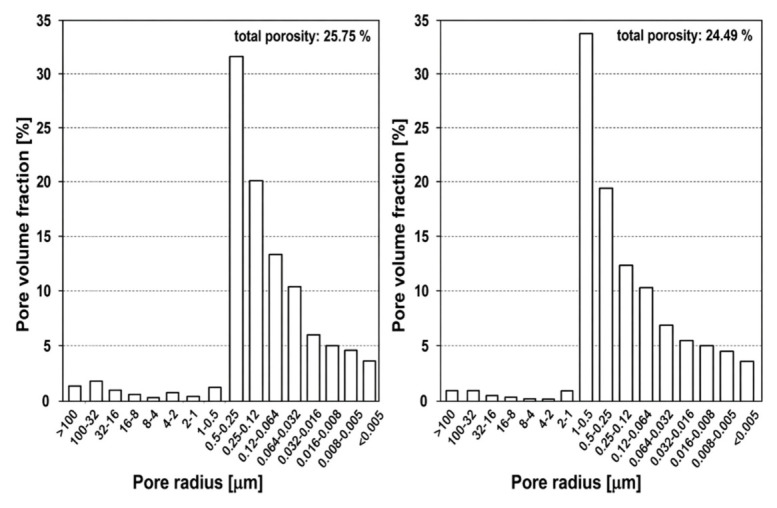
Pore size distribution of earth elements.

**Figure 10:**
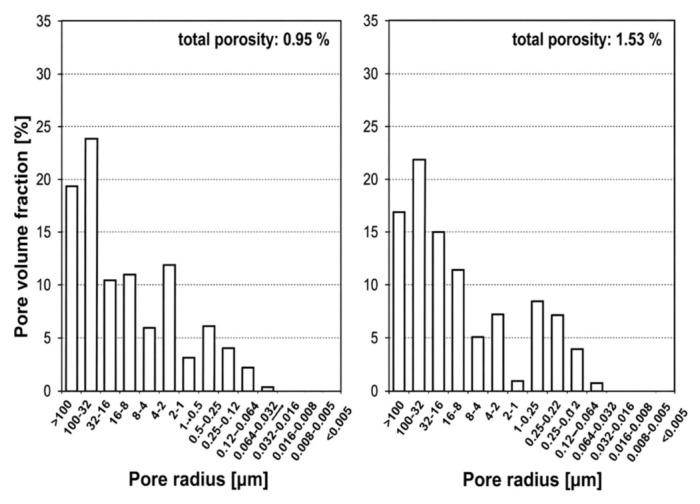
Pore size distribution of stone samples.

The mercury intrusion porosimetry results obtained for both the earthen elements (Figure 9) and the stone samples (Figure 10) demonstrated a high degree of reproducibility and consistency across all tested specimens. This is particularly noteworthy for the clay-based (earthen) materials, given their manual preparation and the expected intrinsic heterogeneity associated with such processing. The observed repeatability indicates that, despite potential local variations introduced during hand shaping, the overall pore structure distribution is sufficiently uniform at the representative volume scale captured by MIP analysis. These findings support the reliability of the measured porosity parameters and validate their use for subsequent correlation with non-destructive testing results. Total porosity of examined model samples is given in Table 5.

### 3.3. Estimated Mechanical Properties of Stone and Clay Samples

Conventional modulus–strength relationships were primarily developed and calibrated for concrete and similar dense, quasi-homogeneous materials. Their direct application to natural stone and unfired clay is therefore not straightforward. In the case of stone, differences in microstructure, including mineral composition and grain interlocking, may lead to deviations from concrete-based correlations. For unfired clay, the discrepancy is more pronounced due to its high porosity, heterogeneity, and significant damping, which fundamentally alter the relationship between stiffness and strength. As a result, the application of conventional models to unfired clay leads to a systematic overestimation of compressive strength, indicating that stiffness alone is insufficient to describe its mechanical behavior.

For stone samples, the static elastic modulus was estimated from the experimentally determined dynamic modulus using two procedures. In the first case, a commonly used empirical conversion was applied (*E*s = 0.83 · *E*d), reflecting the well-established observation that the dynamic modulus is typically higher than the static modulus due to the absence of microcracking effects at low strain levels [23,24,25]. In the second case, the static modulus was calculated using an empirical expression according to the BS EN 1992-1-1:2023 [56], (*E*_s_ = 1.25 · *E*_d_ − 19).

Although the estimation of the static modulus differs between these procedures, the compressive strength was calculated using the modified Lyndon–Balendran relationship. The introduced coefficient represents a scaling of the original formulation, providing a more conservative estimation and improving agreement with experimental observations. The flexural strength was estimated using Equation (6) which represents the ACI 318 relationship. Therefore, the differences in the predicted compressive and flexural strengths arise solely from the estimation of the static modulus, while the modulus–strength relationship itself remains unchanged.

(5) $$f_{c}=0.156{\left(\frac{E_{s}}{5500}\right)}^{3}$$

(6) $$f_{r}=0.62\sqrt{f_{c}}$$

For unfired clay, a similar conceptual framework was adopted; however, due to the absence of standardized modulus–strength relationships, simplified empirical formulations were used. The static modulus was estimated from the dynamic modulus as *E_s_* = 0.40 *E*_d_ and *E*_s_ = 0.55 E_d_, and the corresponding compressive strength was calculated as *f*_c_ = *E*_s_/250 and *f*_c_ = E_s_/150. The flexural strength was estimated using proportional relationships with compressive strength: *f*_r_ = 0.11 *f*_c_ and *f*_r_ = 0.15 *f_c_*. These coefficients represent simplified engineering approximations commonly adopted for porous and heterogeneous materials, where the static modulus is significantly lower than the dynamic modulus due to microcracking and nonlinear deformation effects. These expressions represent simplified engineering approximations, reflecting the reduced stiffness-to-strength ratio in highly porous materials.

The experimentally determined dynamical elastic moduli, estimates of static elastic properties, and mechanical strengths of stone and unfired clay samples obtained by different methods (MARM, IET, and UT) were reported in Table 6 and Table 7. The longitudinal dynamic modulus of the stone sample shows very good agreement among the three methods, with values between 85.17 and 87.21 GPa. Thus, the proposed non-contact MARM technique can be considered reliable and consistent compared to accelerometer-based and ultrasonic methods. The proposed MARM approach additionally enables simultaneous non-contact evaluation of elastic modulus and damping characteristics within a single experimental setup. The bending and torsional moduli, calculated by MARM and IET, also show a close match, with differences consistent; hence, the modal identification is stable, and sensor coupling or excitation differences have a negligible impact. Table 7 for the earthen sample shows significantly reduced elastic moduli and strengths, which visually correspond to the material’s unfired, porous nature. Dynamic moduli from MARM, IET, and UT for these samples are more dispersed than those of the stone, especially for flexural and torsional modes, which is explained by higher damping, more porosity, and microstructural heterogeneity. Static elastic moduli and strength values derived from the engineering approximations exhibit a very strong dependence on the chosen empirical model (standard versus high-quality product), underscoring the crucial role of porosity and an informed interpretation.

The results obtained using these approaches demonstrate significant variability in the predicted strength values for unfired clay, depending on the selected empirical model. This confirms the need for a formulation that explicitly accounts for the material’s porosity and damping characteristics.

The results obtained for stone samples show a very good agreement between the applied non-destructive methods, including MARM, ultrasonic testing, and the accelerometer-based method. The differences in the measured elastic modulus are within a few percent, confirming the consistency and reliability of the applied procedures.

The Poisson’s ratio values obtained using the standard procedures are within the expected range for stone materials, further supporting the validity of the measurements.

In contrast to stone, the results obtained for unfired clay show a higher variability between different methods. This behavior is attributed to the porous and heterogeneous structure of the material, as well as increased damping effects. The IET method yields scattered and, in some cases, unrealistically high Poisson’s ratio values. A sensitivity analysis was performed with respect to the assumed Poisson’s ratio. The results indicate that variations in the assumed value led to only minor differences in the calculated elastic modulus, with deviations typically below 0.02. The raw experimental data, including the full dataset and recorded acoustic signals used in the MARM measurements, are provided in the Supplementary Materials. This confirms that the solution is numerically stable and not highly sensitive to the initial assumption.

This suggests that traditional dynamic testing techniques may not be immediately applicable to very porous materials. Despite the heterogeneity in Poisson’s ratio, the estimated elastic modulus values stay generally steady across multiple approaches, implying that modulus estimation is more reliable than direct determination of Poisson’s ratio.

Based on these observations, a model for compressive strength estimation was developed. In parallel, the intrinsic elastic properties of the material were determined using a Pabst-type modulus–porosity Equation (2), where the exponent *n* was adopted as 2.5 from literature and not treated as a fitting parameter. The parameters *E*_0_ and *G*_0_, representing the longitudinal and torsional moduli of a fully dense material, were obtained by nonlinear least-squares fitting of the experimental data using Excel Solver tool. For unfired clay, the estimated values were respectively 14.84 and 7.01 GPa for *E*_0_ and *G*_0_. If these values are compared to measured *E* and *G* values a reduction in stiffness of about 50% is registered due to the present porosity.

The proposed Equation (7) relates compressive strength directly to the estimated elastic modulus and porosity. Here, E_s,app_ represents the elastic modulus taken from Table 4, which was derived from dynamic measurements, incorporating damping effects through the measured loss factor, while φ is the total porosity. The model is formulated on the basis of measurable parameters and reflects the combined influence of stiffness, pore structure, and energy dissipation on the mechanical behavior of earthen products. It should be noted that damping effects are already incorporated into the estimated apparent modulus *E*_s,app_, which was derived from acoustic measurements. Therefore, the additional coefficient m does not account for energy dissipation, but rather represents the remaining difference between elastic stiffness and compressive strength.

(7) $$f_{c}={mˑE}_{s,app}{\left(1-\emptyset \right)}^{2.5}$$

This highlights that strength is not directly proportional to the measured modulus alone, but is governed by the combined effect of intrinsic stiffness and porosity. While E_0_ does not directly represent strength, it defines the upper-bound stiffness of the solid phase and thus the mechanical potential of the material. The results confirm that porosity is the dominant factor controlling strength reduction, whereas the intrinsic modulus governs the limiting behavior of the material.

The proposed formulation should therefore be interpreted as a porosity-sensitive engineering approximation rather than a universal constitutive relationship. The coefficient (m) was introduced as a calibration parameter accounting for microstructural effects not fully captured by elastic stiffness and porosity alone, including pore connectivity, stress concentration, and energy dissipation mechanisms. The obtained results indicate that the proposed approach provides more realistic estimations for highly porous materials such as unfired clay, whereas larger deviations may occur for low-porosity materials. Consequently, the applicability of the proposed model is currently limited primarily to highly porous material systems.

For unfired clay, the calibrated coefficient m was on the order of 10^−3^. This coefficient represents a correction factor accounting for microstructural effects that are not fully captured by elastic modulus and porosity alone. In contrast to conventional empirical relationships, the proposed model does not rely on predefined modulus–strength correlations. Instead, it is derived from experimentally observed trends and provides a more realistic estimation of compressive strength for highly porous materials. The results obtained for stone samples primarily serve as validation of the applied non-destructive testing methods. In contrast, the behavior of earthen samples indicates that conventional modulus–strength relationships are not directly applicable, motivating the development of a dedicated strength estimation model. Table 8 presents the comparison between the uncalibrated model (*m* = 1) and the calibrated formulation. The uncalibrated model slightly overestimates the compressive strength of unfired clay, while the introduction of the coefficient m significantly improves the agreement with experimental values.

The proposed model is not applicable to dense materials such as stone, as it leads to significant overestimation of compressive strength. The flexural strength estimated using proportional relationships shows better agreement with experimental values when the uncalibrated compressive strength is used. This suggests that flexural behavior is less sensitive to the applied correction factor and follows the general stiffness–porosity trend. For earthen samples, the flexural strength estimated using proportional relationships yields values of 0.38 MPa (0.11·*f*_c_) and 0.52 MPa (0.15·*f*_c_) when the uncalibrated compressive strength is used. These values are closer to the experimentally measured flexural strength (0.63 MPa) compared to those obtained from calibrated compressive strength, indicating that flexural behavior is less sensitive to the applied correction factor.

When the uncalibrated model (*m* = 1) is used, the predicted compressive strength slightly overestimates the experimental value, while the corresponding flexural strength shows improved agreement. This indicates that the proposed formulation provides a reasonable engineering approximation of the overall mechanical behavior of the material. By introducing the coefficient m, the compressive strength prediction is significantly improved, achieving very good agreement with experimental results. The parameter m can therefore be interpreted as a correction factor accounting for microstructural effects not captured by elastic stiffness and porosity alone. Results are given in Supplementary File. These findings highlight that compressive and flexural strengths are affected differently by porosity and microstructure. While compressive strength is more sensitive to internal defects and requires calibration, flexural strength appears to be better approximated using simplified proportional relationships once the global stiffness–porosity trend is captured.

Detailed calculation procedures, including full datasets and intermediate steps for modulus determination, are provided in the Supplementary Materials.

## 4. Conclusions

This study investigated the applicability of non-destructive testing methods for the characterization of mechanical properties of stone and unfired clay materials using the modified acoustic resonance method (MARM), the impulse excitation technique (IET), and ultrasonic testing (UT). The obtained results demonstrated very good agreement between the applied methods for stone samples, confirming the reliability of non-destructive approaches for dense and relatively homogeneous materials. In contrast, unfired clay exhibited increased variability between methods due to its porous and highly dissipative microstructure.

The main contribution of this study is the development and application of a modified acoustic resonance method based on a two-microphone non-contact setup capable of simultaneously determining dynamic elastic modulus and damping characteristics within a single experimental procedure. The study further demonstrated that damping represents an important parameter in the interpretation of acoustic measurements, particularly for highly porous materials such as unfired clay. The proposed methodology enables completely non-invasive evaluation of elastic and dissipative behavior without the need for sensor attachment or destructive sampling.

The results also showed that conventional modulus–strength relationships are not directly applicable to unfired clay, as they significantly overestimate compressive strength. To address this limitation, a simplified engineering approach based on estimated elastic modulus and porosity was introduced. The obtained results indicate that the proposed formulation provides realistic estimations primarily for highly porous materials, while its applicability to dense materials remains limited. Finally, the presented methodology provides a consistent framework for the non-destructive evaluation of porous and fragile materials relevant to cultural heritage research.

## Figures and Tables

**Table 1:** Experimentally measured and numerically predicted resonance frequencies for the stone sample (longitudinal *f*_L_, flexural *f*_F_, and torsional *f*_T_ modes).

Condition	Stone Sample 4			Earth Element Sample 3		
	*f_L_* (Hz)	*f_F_* (Hz)	*f_T_* (Hz)	*f_L_* (Hz)	*f_F_* (Hz)	*f_T_* (Hz)
Numerical, 1st iteration (*E* = 60.00 GPa, *ν* = 0.23)	7728	2317	4525	7522	3167	4385
Numerical, 2nd iteration (*E* = 85.17 GPa, *ν* = 0.27)	9203	2759	5305	6819	2872	4005
Measurement	9190	2586	5227	6770	2894	3845

**Table 2:** Density and geometric characteristics of tested samples.

Property	Stone Samples	Earth Element Samples
Density (kg/m^3^)	2777	1917
Length (m)	0.300	0.147
Width (m)	0.050	0.037
Thickness (m)	0.045	0.037

**Table 3:** Average resonant frequencies determined by MARM and IET.

Average Value	Stone Samples		Earth Element Samples	
	MARM	IET	MARM	IET
*f_L_* (Hz)	9225	9574	6775	6583
*f_F_* (Hz)	2634	2550	2921	2485
*f_T_* (Hz)	5278	5346	3978	3308

**Table 4:** Damping parameters and dynamic elastic moduli for model samples.

Property	Stone Samples			Earth Element Sample		
*f* (Hz)	9190	2586	5227	6770	2894	3845
*T*_10_ (s)	0.088	0.274	0.067	0.006	0.019	0.014
*η*	0.0027	0.0031	0.0063	0.0542	0.0400	0.0409
*E_d_* (GPa)	82.39			7.33		
*E_l_* (GPa)	0.26			0.29		
*E_s,app_* (GPa)	82.23			7.14		

**Table 5:** Total porosity of examined model samples.

Sample	Stone Models		Earth Element Models		
Measured total porosity	Stone 1	Stone 2	Earth element 1	Earth element 2	Earth element 3
	1.53	0.95	25.75	24.49	25.39
Average values	1.24		25.21		

**Table 6:** Estimated mechanical properties of stone (validation of standard models).

Dynamic Elastic Moduli (Measured)			
Property	MARM	IET	UT
Longitudinal modulus, *E*_L_ (GPa)	85.17 ± 0.75	87.21 ± 1.58	86.36 ± 1.18
Flexural modulus, *E*_F_ (GPa)	82.39 ± 2.27	77.23 ± 2.02	—
Torsional modulus, *E*_T_ (GPa)	33.57 ± 0.45	34.44 ± 0.77	—
Estimated Static Elastic Modulus			
Approach	MARM	IET	UT
Empirical (in GPa)	70.69	72.38	71.68
BS EN 1992-1-1:2023 (in GPa)	72.39	74.13	73.41
Estimated Compressive Strength			
Scaled Lyndon–Balendran (in MPa)	331.81	356.18	345.88
	361.98	370.64	367.03
Average–Approach 1 (in MPa)	344.62		
Average–Approach 2 (in MPa)	366.55		
Estimated Flexural Strength			
According to ACI 318 (in MPa)	26.54	28.49	27.67
	28.96	29.65	29.36
Average–Approach 1 (in MPa)	27.57		
Average–Approach 2 (in MPa)	27.64		
Experimental Reference Values			
Flexural strength (MPa)	23.50 ± 2.60		

**Table 7:** Estimated Mechanical Properties of earth elements (model sensitivity).

Dynamic Elastic Moduli (Measured)			
Property	MARM	IET	UT
Longitudinal modulus, *E*_L_ (GPa)	7.64 ± 0.70	7.21 ± 0.66	6.69 ± 0.91
Flexural modulus, *E*_F_ (GPa)	7.33 ± 0.50	5.31 ± 0.41	—
Torsional modulus, *E*_T_ (GPa)	3.12 ± 0.31	2.16 ± 0.22	—
Estimated Static Elastic Modulus			
Model	MARM	IET	UT
Empirical model M1standard product quality (GPa)	3.05	2.88	2.68
Empirical model M2 high product quality (GPa)	4.20	3.96	3.68
Estimated Compressive Strength			
Conversion of (M1) (MPa)	12.22	11.53	10.70
Conversion of (M2) (MPa)	28.00	26.43	24.53
Estimated Flexural Strength			
Conversion of M1 (MPa)	1.34	1.27	1.18
Conversion of M2 (MPa)	4.20	3.96	3.68
Averaged M1 (MPa)	1.26		
Averaged M2 (MPa)	3.95		
Experimental Reference Values			
Flexural strength (MPa)	0.627 ± 0.11		
Compressive strength (MPa)	2.56 ± 0.63		

**Table 8:** Application of the proposed model (calibrated and uncalibrated).

Material	$E_{s,app}$ (MPa)	∅	${\left(1-\emptyset \right)}^{2.5}$	$f_{c}$ (m = 1) (MPa)	m	$f_{c}$ (Calibrated)	$f_{c}$ (Exp) (MPa)
Stone	82,230	0.0124	0.969	79,600	—	—	345–366
Unfired clay	7140	0.2521	0.486	3470	0.00074	2.56	2.56

## Data Availability

The original contributions presented in this study are included in the article/Supplementary Materials. Further inquiries can be directed to the corresponding author.

## Supplementary Materials

The following supporting information can be downloaded at: https://www.mdpi.com/article/10.3390/ma19112291/s1.

## Abbreviations

MARM	A modified acoustic resonance method
IET	Impulse excitation technique
UT	Ultrasonic pulse velocity testing
*f*_L_	Longitudinal mode
*f*_F_	Flexural mode
*f*_T_	Torsional mode
E_d_	Dynamic elastic modulus
E_s_	Static elastic modulus
E_s,app_	Estimated modulus including damping
φ	Porosity
f_c_	Compressive strength
f_f_	Flexural strength
